# Allergy Takes its Toll: The Role of Toll-like Receptors in Allergy Pathogenesis

**DOI:** 10.1097/wox.0b013e3181625d9f

**Published:** 2008-01-15

**Authors:** Susan L Prescott

**Affiliations:** 1 School of Paediatrics and Child Health, University of Western Australia, Princess Margaret Hospital for Children, GPO Box D184, Perth, Western Australia 6840

**Keywords:** toll-like receptors, allergic disease, "hygiene hypothesis", cord blood, childhood, cytokines, allergy prevention

## Abstract

Reduced early microbial exposure in early life has become a leading candidate to explain the escalating rate of allergic disease and has generated intense interest in the interaction between the developing immune system and the microbial environment. Infants depend on "signal" from the microbial environment to mature both T-helper cell type 1 and regulatory immune function. These signals, mediated through microbial pattern-recognition receptors, namely toll-like receptors (TLRs), seem essential to achieving the immunologic balance required for (1) pathogen protection and (2) normal immune tolerance. Despite this, the normal development of TLR function has never been documented. There is preliminary evidence that TLR function is under the influence of both genetic factors (genetic polymorphisms) and early environmental exposures including microbial exposure and breast feeding, and maternal smoking. This review explores the evidence that allergy is associated with developmental variations in TLR-mediated immune function and how this may be relevant for disease prevention.

## 

The epidemic rise in asthma and allergic disease during the last 40 years highlights the urgent need to identify the developmental pathways that lead to disease. Although this rise in allergic disease is likely to be multifactorial, one of the most plausible candidates has been the apparent decline in microbial burden in early life. A better understanding of the links between innate immune and cognate (allergen-specific) immune development is the logical next step in the quest to define allergy pathogenesis. There is escalating interest in the pathways that influence maturation of the key cells involved in early immune regulation, namely antigen-presenting cells (APCs) and regulatory T cells. These cells depend on toll-like receptor (TLR)-mediated signaling for activation and maturation, and there is growing speculation that both genetic polymorphisms (affecting TLR function) and environmental factors (such reduced microbial exposure) predispose to allergic disease through effects on innate immune pathways. Better understanding of the development of these pathways is also likely to contribute to more avenues for better targeted treatment and prevention.

This review explores the evidence to support the emerging hypothesis that:

1. There is normal age-related maturation of TLR-mediated innate immune pathways, and genetic predisposition and early environmental exposures result in individual variations in the rates of maturation and functional patterns;

2. Altered maturation in TLR-mediated responses are associated with effects on antigen-specific immune responses (including increased predisposition for T-helper cell type 2 (T_h_2) allergic responses to allergens); and

3. Children who develop allergy show differences in early maturation of TLR-mediated responses.

## The role of microbial stimulation for early immune development

The current paradigm maintains that immature T_h_2-dominant neonatal responses must undergo environment-driven maturation in the early postnatal period, [1,2] with gradual inhibition of this early T_h_2 propensity in favor of more mature T_h_1 dominant responses. Although the responsible factors are still unclear, experimental models [3] have provided strong support for the hypothesis that early microbial exposure is critical for normal development (reviewed in Strachan [4] and Prescott [5]). As the most powerful immunostimulants in the normal environment, these agents generally activate the innate immune system to promote host defense through T_h_1 effector function.

## The role of TLR pathways in immune regulation

Toll-like receptors are essential to first-line innate defense mechanisms that have evolved to recognize bacteria, viruses, and other microorganisms in the potential absence of established immunologic "memory." This family of receptors recognize a broad range of microbial agents [6] with different TLR signaling the presence of different micobial components. The TLRs are found on many cells involved in immediate host defense such as neutrophils, natural killer cells, and APCs, which also play a critical role in programming subsequent adaptive T-cell responses. Once activated via these TLR pathways, dendritic cells and other APCs show enhanced expression of costimulatory molecules and cytokines (including interleukin 12 [IL-12]), which favor T_h_1 immune differentiation. More recently, TLRs (TLRs 4, 5, 7, and 8) have also been identified on CD4+ CD25+ T-regulatory cells [7] that play a critical role in controlling immune responses [8]. Thus, it has been proposed that TLR-mediated activation of both APC and regulatory T cells may play an important role in reducing the risk of T_h_2-mediated allergic responses (Wills-Karp et al, [8] Holt et al, [9] and others). This is obviously of most relevance in early life when programming of immunologic function is initiated. Despite this, the ontogeny of TLR function has not been documented.

There is growing speculation that TLR function matures in the postnatal period and that children who develop allergic disease have differences in these development patterns as a result of genetic predisposition and/or environmental influences. This hypothesis is based on preliminary evidence that:

a. TLR function is developmentally regulated, with differences between infants and adults; and that

b. genetic and environmental factors can modify TLR function in early life, as discussed later.

## Preliminary evidence that TLR function is developmentally regulated

**Figure 1:**
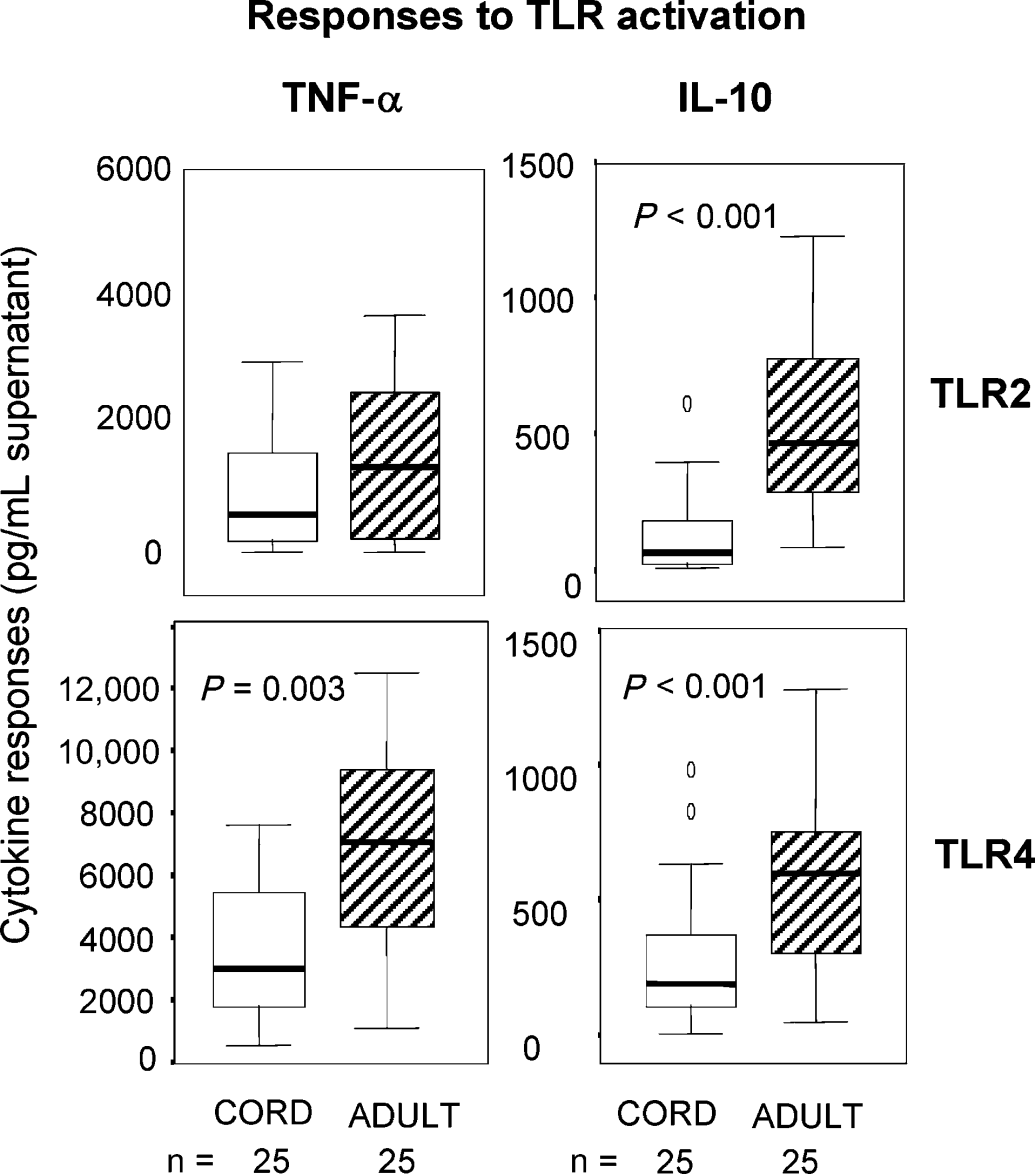
**Comparison of neonatal and adult responses to TLR activation: TNF-*α *and IL-10 responses were measured after stimulation with a TLR2 ligand (pansorbin) and TLR4 ligand (LPS)**. The cytokine levels in culture supernatants (picograms per milliliter) after 48 hours are displayed as median, and 5, 25, 75, and 95 percentile ranges and outlying values. All responses are shown as the levels in stimulated cultures after subtracting levels in the unstimulated control cultures. Data from adults (shaded bars) were compared with neonates (unshaded bars) using nonparametric Mann Whitney *U *test (with significance values as shown).

Although there are no longitudinal studies of TLR development in the early postnatal period, our preliminary studies show significantly lower TLR-mediated responses in neonates (cord blood) compared with adults (as shown on Figure 1). In these studies, we used TLR2 (pansorbin) and TLR4 (lipopolysaccharide [LPS]) ligands to activate these innate pathways, with cytokine detection as a measure of pathway function. The production of inflammatory cytokine (tumor necrosis factor-*α *[TNF-*α*]) and regulatory cytokine (IL-10) was attenuated in the neonates. This was also seen for IL-6 (*P *= 0.045, not shown). This is consistent with other reports demonstrating differences in TLR function between neonates and adults [10,11]. We have also reported functional differences in neonatal responses to TLR9 ligands (microbial cytosine-phosphate-guanine [CpG] motifs) compared with adults [12]. Finally, others have shown age-related increases in the levels of circulating soluble (s)CD14, which is involved in TLR4 signaling [13]. Together, these observations strongly support the hypothesis that TLR responses mature in the postnatal period.

## Preliminary evidence that TLR function is influenced by early events

There is also emerging evidence that TLR function (and maturation of function) is regulated by both genetic and environmental factors.

### Evidence that TLR function is influenced by genetic factors

**Figure 2:**
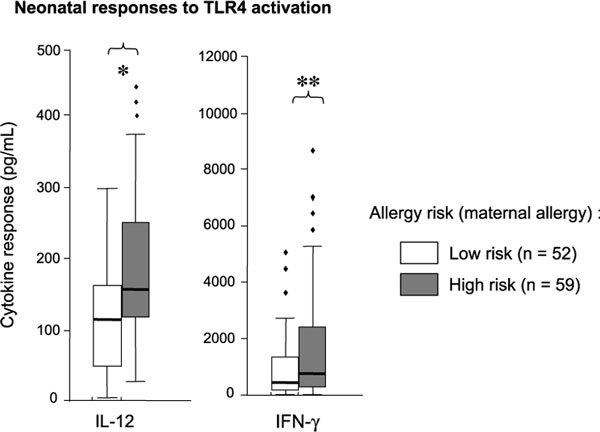
**Comparison of TLR4-mediated responses in neonates at high and low risk of allergy (based on maternal allergy): interferon-γ and IL-12 responses were measured after stimulation with TLR4 ligand (LPS)**. The cytokine levels in culture supernatants (picograms per milliliter) after 48 hours are displayed as median, and 5, 25, 75, and 95 percentile ranges and outlying values. All responses are shown as the levels in stimulated cultures after subtracting levels in the unstimulated control cultures. Data from high-risk neonates (shaded bars) were compared with low-risk neonates (unshaded bars) using nonparametric Mann Whitney *U *test. Significance values are shown as *p < 0.01; **p < 0.05.

Genetic polymorphisms in TLR have been shown to confer variability in functional responses. The TLR4 polymorphisms have been associated with LPS hyporesponsiveness [14] and variations in LPS-induced IL-12 and IL-10 responses [15]. Familial allergy risk (maternal allergy) has also been associated with reduced neonatal TLR2 responses (*p *= 0.03; n = 9), and lower TLR2, TLR4, and CD14 messenger RNA levels in cord blood (n = 185) [16-18]. This has led to speculation that inherited predisposition to TLR immaturity may contribute to less effective TLR-mediated inhibition of allergic responses. However, findings of a recent cohort study by our group contradict this. Instead, we observed that high-risk infants (n = 59) had significantly higher (rather than lower) responses to TLR2, TLR3, and TLR4 ligands (particularly IL-12 responses) than low-risk infants (n = 52; shown for TLR4 on Figure 2) (M. Tulic, P. Noakes, B. Chow, et al., unpublished data, 2008). Although the relevance of this is not clear, these observations do suggest that genetic factors may lead to differential expression and function of these pathways.

### Evidence That TLR Function Can Be Altered by Early Environmental Exposures

Microbial exposure is likely to be a major factor influencing TLR function. Intrauterine infection has been associated with increased placental TLR(4) expression, [19] however, the effects of variation in ambient microbial exposure are less clear. Some studies suggest that TLR expression (level of receptor detected) is related to maternal LPS exposure pregnancy, [20] whereas others show that high exposure is associated with significant down-regulation of responses to TLR ligands (LPS) that were notably also associated with less allergic disease and sensitization [21]. These apparently divergent findings could relate to differences in measuring levels versus function. Although more studies are needed to determine the significance, these findings do suggest that early microbial exposure can modify innate receptor expression and function.

A number of other environmental exposures have been associated with TLR expression. Human milk (as opposed to formula milk) has been associated with differential TLR expression (with down-regulation of TLR2 and TLR3 function and up-regulation of TLR4 and TLR5) [22]. The relationship between these immunomodulatory effects and the immunopro-tective properties of breast milk are not yet clear. Recent studies also indicate that the immunomodulatory properties of n-3 polyunsaturated fatty acids (fish oil) are mediated, at least in part, through TLR4 [23]. Finally, we have recently shown that specific exposures in pregnancy (such as maternal smoking) are associated with significantly reduced TLR function [24]; with effects on TLR2, -3, -4, and -9 function [25]. There were no effects with other (non-TLRYmediated) methods of stimulation (such as phytohemagglutinin mitogen), suggesting selective effects on TLR signaling (possibly through common signal transduction pathways such as activator protein 1 or nuclear factor-κB.

Together, these observations suggest that TLR function is influenced by both genetic and environmental exposures in early life, providing a strong basis for our hypothesis that these variations will have effects on subsequent immune development.

## Evidence that variations in TLR function could influence development of allergen-specific responses or the risk of allergic disease

Although this has not been examined directly in humans, genetic studies provide supportive evidence that functional variations in aspects of TLR microbial signaling are associated with clinical phenotypes and disease. Specifically, polymorphisms in the gene coding for CD14 have been linked to total serum immunoglobulin E levels, [25] suggesting functional relationship between atopy and TLR4 pathways. Some more recent studies have shown that TLR4 genetic polymorphisms have a protective effect on asthma, [15] but others have not [26,27]. The TLR2 genetic polymorphisms have also been shown to have a protective effect on asthma [28]. Notably, the protective effect was only seen when children were raised in environments with "high" microbial burden, illustrating the interactive effects of genetic and environmental factors on these pathways. Together, these findings suggest that alterations in TLR function, either as a result of differences in early environmental exposures or functional genetic polymorphisms, have an effect on subsequent development of adaptive immune function.

As part of our recent TLR studies, we compared neonatal TLR function according to subsequent allergic outcomes in the first year of life (diagnosed as food allergy or atopic dermatitis), and noted that the nonallergic group (n = 74) had lower TLR-mediated responses than the allergic group (n = 28) [19]. These were most notable in response to TLR3 ligands (significantly lower TNF-*α *and IL-12 responses) and TLR5 ligands (significantly lower TNF-*α *and IL-10 responses), although similar trends were seen with other ligands. This seemingly paradoxical observation that lower TLR responses are protective is consistent with observations that the protective effects of microbial exposure are also associated with down-regulated TLR-mediated microbial responses [22]. The significance and mechanisms of these relationships remain to be determined. Thus, whereas alterations in TLR function seem to be associated with the development of allergic disease, the initial hypothesis (that reduced TLR function predisposes to allergic disease) seems oversimplistic.

## Evidence that TLR activation could be a way to reduce the risk of allergy

A number of studies suggest that early microbial-driven TLR activation may modify immune development and the risk of sensitization. In an animal model, Blumer et al [29] recently demonstrated that antenatal (maternal) exposure to bacterial LPS enhanced neonatal T_h_1 interferon-γ responses and inhibited (ovalbumin) allergen sensitization in the offspring. This demonstrates that immune activation via the TLR4 pathway can modify allergen-specific immune development. Tulic et al [30] have shown similar effects of this TLR4 ligand (LPS) in the postnatal period, but notably, the inhibition of allergic responses was seen only when LPS was given before responses were established. There is only indirect evidence that early bacterial exposure may be protective in humans. This includes the many epidemiological studies (reviewed by Strachan [4]) that formed the basis of the "hygiene hypothesis." Intervention studies also suggest that administration of bacterial products to children may have clinical (Arkwright and David, [31] Weston et al, [32] and others) and immune effects [33]. Although it has been inferred that these variations in microbial exposure may be responsible for differences in innate (and subsequent cognate) immune function, this has not been documented directly.

The main intervention studies that use microbial products to prevent allergic disease in humans have used nonpathogenic probiotic species. At least some of the anti-inflammatory effects seem to be mediated through TLR, including TLR9 [34] and possibly TLR2 and TLR4 expressed on enterocytes. These flora have been shown to promote regulatory populations [35] and tolerogenic dendritic cells [36-38] to promote T_h_1 differentiation [39]. Initial clinical studies showed promising results in the prevention of allergic disease (although this was limited to atopic dermatitis), [40] however, not all subsequent studies have shown a benefit [41,42]. This may be caused by differences in the species, dose and timing of supplementation, and other host and environmental factors. It remains likely that microbial products could have a role in disease prevention, although more targeted and more refined strategies may need to be developed before this is possible. A better knowledge of TLR ontogeny and the effects of functional genetic polymorphisms on treatment response will be essential to this process.

In summary, infants seem to depend on signals from the microbial environment to mature both T_h_1 and regulatory immune function. These signals, mediated through pattern-recognition receptors such as TLR, seem essential to achieving the immunologic balance required for (1) pathogen protection and (2) normal immune tolerance. It is therefore highly logical to further investigate the potential role of impaired TLR signaling in the development of allergic disease. The current evidence supports the notion that genetic and environmental factors can result in early variations in TLR function, and that this can affect adaptive immune development. Further studies are needed to assess normal ontogeny of TLR function and to assess differences in atopic infants. This should lead to a better understanding of the role of bacterial products in prevention or early treatment, and how genetic factors may influence individual differences in responsiveness to potential intervention strategies.

## Notes

Supported by the National Health and Medical Council of Australia. Studies on toll-like receptors are funded by the National Health and Medical Research Council of Australia
